# Cosmetic Syndrome Correction with Calcium Hydroxylapatite-Based Filler in Patients with Connective Tissue Dysplasia

**DOI:** 10.1155/2021/6673058

**Published:** 2021-04-14

**Authors:** Maria Shirshakova, Elena Morozova, Daria Sokolova, Svetlana Pervykh, Lyailya Kayumova

**Affiliations:** ^1^Aesthetic Clinics of Maria Shirshakova, Moscow, Russia; ^2^I. M. Sechenov First Moscow State Medical University (Sechenov University), Moscow, Russia; ^3^Clinic: Center of Medical Practice, Moscow, Russia; ^4^Clinic of Evidence-Based Medicine DocMed, Moscow, Russia

## Abstract

Undifferentiated connective tissue dysplasia is one of the most common diseases of nowadays, which does not fit into the group of hereditary syndromes. This condition is diagnosed in 20–50% of the population at any age. The study aimed to correct the facial soft tissues of patients with undifferentiated connective tissue dysplasia through the cosmetic procedure of calcium hydroxylapatite injection. In 2018, a 36-year-old patient addressed the beauty salon with signs of undifferentiated connective tissue dysplasia, such as severe asymmetry of the face, infraorbital and nasolabial sulci, and thin and easily folding skin. Signs were observed from the age of 22, i.e., for 14 years. The therapy was performed using special features of the correction of facial soft tissue changes in patients with connective tissue dysplasia (CTD) using calcium hydroxylapatite-based products (Radiesse®, Merz North America, Inc., USA). Particular attention is given to the need for early correction to prevent premature skin aging related to this condition. After 14 days, a significant improvement of the patient's skin condition was noted after the passing of two procedures. Her condition was estimated as consistent with T1-2P0G0A1Zh1 P1M1K1 and corresponded to grade I age-related changes in the superficial soft tissues. The performed treatment showed high efficacy in case of mild connective tissue dysplasia diagnosis. The results showed that when collecting information from anamnesis, the diagnostic criteria for dysplasia should be considered. If the criteria are met, the cosmetological correction with collagen stimulators becomes possible.

## 1. Introduction and Literature Review

Connective tissue is one of the most widely represented tissues in the human body. In total, connective tissue builds up to 50–80% of body weight. Dense connective tissue forms the skin (which, in turn, refers to the largest organs of the human body), as well as ligaments, tendons, and fascia. The loose connective tissue contributes to the formation of the stroma in various organs. Connective tissue consists of dentin and enamel in the teeth, synovial and serous membranes, and cornea and crystalline lens. Thus, connective tissue constitutes nearly all systems and organs of the human body, performing such important functions as protector and barrier, as well as actively participating in metabolic processes of the body.

Due to the widely expressed polymorphism of connective tissue and its participation in numerous processes in the body, a large number of disorders in its functioning emerge, acquired by a person genetically or relative to the impact of adverse environmental factors. These disorders may contribute to the development of secondary pathologies of the organs and their systems, as well as dysplastic-dependent diseases associated with clinical and morphological disorders in the work of connective tissue. Further progression of these diseases leads to the emergence and strengthening of chronic diseases. The main cause of most diseases is dysplasia of connective tissue, acquired in adolescence.

### 1.1. Characteristic Features of Connective Tissue Dysplasia

Connective tissue dysplasia is a widespread disease among the population, and the frequency of which can be within 7–10% (data for the Russian Federation) [1]. Most often, this disease develops in regions with the concentration of zinc and magnesium below normal, in Udmurtia (Russia), and it is fixed in 30–70% of adolescents. It follows that dysplasia prevails at a young age and can lead to a violation of social adaptation of people and reduction of quantitative and qualitative indicators of birth rate and working capacity of the population. Thus, dysplasia of connective tissue can have a wide range of negative effects on physical and mental health. The research efforts aimed at studying dysplasia is still relevant today. The measures taken to prevent dysplasia include early diagnosis of dysplasia, drug treatment, and rehabilitation therapy procedures [2].

Connective tissue dysplasia (CTD) is a genetically determined condition characterized by defects in the fibrous structures and the ground substance of the connective tissue and resulting in the impaired formation of organs and organ systems. CTD is characterized by a progredient course, which defines characteristic features of the related pathology, as well as pharmacokinetic and pharmacodynamic properties of the medicinal products [3]. Various forms of differentiated CTD are known for over 100 years, which include Marfan syndrome, Ehlers–Danlos syndrome, and osteogenesis imperfecta (all included in the ICD) [4].

In terms of morphology, CTD is characterized by changes in collagen and elastic fibrils, glycoproteins, proteoglycans, and fibroblasts. These changes are determined by inherited mutations in the genes encoding the synthesis and spatial organization of collagen, structural proteins, and protein-carbohydrate complexes, as well as certain enzymes and their cofactors [5]. As a result of the impaired formation of collagen chains, the so-called abnormal collagen trimers are formed, which do not resist mechanical loading. CTD has multiple phenotypic variants depending on the end organs involved.

The key signs of connective tissue dysplasia suggesting the presence of this pathology include the following [6]:

- Prolapse of heart valves (mitral valve prolapse is most common, 70%)
- Chest and spine deformation
- Varicose veins of the upper and lower extremities, hemorrhoidal veins, etc.
- Ptosis and dystopia of internal organs
- Myopia, astigmatism, hyperopia, and strabismus
- Flat feet
- Depression and hypochondria

The above individual signs are not strictly specific for CTD and require clinical evaluation and differential diagnosis.

## 2. CTD-Related Cosmetic Syndrome: Main Manifestations

In this article, the skin changes corresponding to the cosmetic syndrome in patients with CTD are discussed in detail. This disease occupies third place in the frequency of clinical manifestations. Cosmetic syndrome is formed throughout the period from birth to the end of growth. Signs of CTD-related cosmetic syndrome are as follows:

- Thin, fragile, sluggish, dry, stretchy (more than 3 cm) skin
- Atrophic stretch marks (striae)
- Healing in the form of wide atrophic scars with a thin appearance often described as being “like tissue paper”
- Keloid scars
- Easy bruising, formation of ecchymoses and petechiae
- Dysplasia-dependent dysmorphic disorders in the maxillofacial area (bite abnormalities, high-arched palate, pronounced facial asymmetry)
- Early appearance of wrinkles and folds
- Premature aging

As noted, children with connective tissue dysplasia form at-risk group for atopic dermatitis with the most severe course [7]. When the above complaints are found in patient's history, the time of the manifestations onset (in childhood or adolescence) and their presence in familial history should be specified [8]. Most often, the skin looks thin, fragile, translucent, and dry; its structure is sluggish and loose, and atrophic stretch marks (striae) may be present. Moreover, some developmental abnormalities can be found in CTD patients (Table 1).

Capillary fragility tests (pinch test, tourniquet test) are positive—bruises, ecchymosis, and petechiae. Due attention should be paid to such a symptom as cutaneous hyperextensibility (painless skin pull (3 cm) on the back of the hand, forehead, elbow joints, lateral ends of the clavicles, and skinfold on the tip of the nose). The type, location, depth, and severity of wrinkles are assessed using the Lemperle clinical and visual scales. These tools are predominantly used to assess severe nasolabial folds and nasolacrimal sulci and are rarely used to assess periorbital and forehead wrinkles [8].

High aesthetic requirements for personal appearance, especially at a younger age, often lead to psychological discomfort and social disadaptation. A proposed examination plan for CTD patients should, therefore, include consultations with various specialists as indicated, i.e., dermatovenerologists, cosmetologists, and plastic surgeons [4]. At the cosmetologist's visit, therapeutic measures aimed at the prevention and correction of age-related skin changes should be recommended as early as possible to patients with deep nasolabial folds and nasolacrimal sulcus because according to the studies, aging manifestations quickly aggravate with CTD. Preventive cosmetic measures can slow down their progression, improve the appearance in patients, and provide high satisfaction of patients.

Early diagnosis of dysplasia includes clinical genealogy methods and detailed analysis of the patient's and family's anamnesis [10]. Another group of methods is biochemical and molecular genetic examinations [11]. Primary methods, however, are those that recognize early stages of dysplasia based on the patient's phenotype, as they are less expensive and require a shorter time interval. These include body mass index assessment, joint hypermobility tests, and the presence of small anomalies (or stigma) [12]. Drug therapy is mainly focused on normalizing the processes of the biosynthesis of collagen and other components in connective tissue, as well as stabilizing metabolic processes in the body [13].

## 3. Methods of Cosmetic Syndrome Correction in CTD Patients

Certain nutritional deficits contribute essentially to the development of CTD. First, these are B vitamins (B1, B2, B3, and B6), which normalize protein metabolism, and vitamins C and E, which support normal collagen synthesis and have an antioxidant effect. Furthermore, a pathogenetic role in the formation of main CTD symptoms has been attributed to copper, zinc, and magnesium deficiencies [3]. A statistically significant decrease in the blood and saliva concentrations of magnesium was detected in patients with undifferentiated CTD and external skin changes [9, 14].

The lack of these micro- and macroelements has been established to increase the degradation of collagen and elastin fibers, as well as polysaccharide fibers of hyaluronan, resulting in lower tensile strength of the connective tissue [15]. Vitamin D has also critical effects on the skin condition and reparative abilities of the connective tissue; its content has a fundamental importance in wound healing and rehabilitation after surgical interventions, peels, mesotherapy, etc. [8].

There is a clear need to eliminate the deficiency of these elements in CTD patients. Once early signs of the cosmetic syndrome appear, a cosmetologist should be consulted as soon as possible in order to correct facial soft tissue changes and to improve skin quality. The first-line cosmetic care includes stimulators of collagen synthesis since the changes in type IV collagen are known to cease the pathological progress [16]. Intradermal injections of 1% hyaluronic acid with various amino acids (proline, lysine, glycine, and cysteine), vitamin C, glutathione, and succinic acid are indicated for the correction of glycosaminoglycan synthesis and improvement of collagen synthesis [8]. Massage or microcurrent therapy can be added to improve microcirculation and tissue trophism.

Administration of calcium hydroxylapatite- (CaHA-) based product is an effective and low-impact method of stimulation of the reticular dermis remodeling and collagen synthesis for correction of facial soft tissues, the formation of solid “matrix,” “basis for ligaments,” and for prevention of their sagging observed in this pathology. According to clinical study data, CaHA-product has been well-tolerated in patients and provided long-term efficacy which is superior to that of hyaluronic acid-based products [17]. Given the aforementioned, a clinical case of the CTD patient subjected to the correction of facial soft tissues changes was described.

### 3.1. Relevance and Aim of the Study

Among the works devoted to dysplasia, the direction of the treatment of this disease and analysis of metabolic disorders occurring in the body prevails [2, 18, 19]. Part of the studies is devoted to the genetic aspect like studying its emergence concerning hereditary factors [20]. Some works are devoted to the psychological aspect of connective tissue dysplasia like changes in the psyche of patients and attitudes to the bodies in persons of different age and gender groups [1]. However, besides clinical and psychological studies, there is a need to examine dysplasia, especially its undifferentiated form, from the standpoint of cosmetology as many patients with this diagnosis primarily appeal to a cosmetologist, setting the task to hide or correct defects in appearance.

A few research efforts in this direction have been attempted [5], which determined the relevance of this article. The effectiveness of the calcium hydroxyapatite administration method is shown on the example of a clinical case. The authors assume that the performed cosmetological procedure will lead to the successful correction of the patient's soft tissues. The results of this report can be used as a basis for similar studies in the future. The purpose of this study was to correct the soft tissues of the patient's face using a cosmetological procedure of calcium hydroxylapatite injection.

## 4. Materials and Methods

### 4.1. Case Report

**Figure 1:**
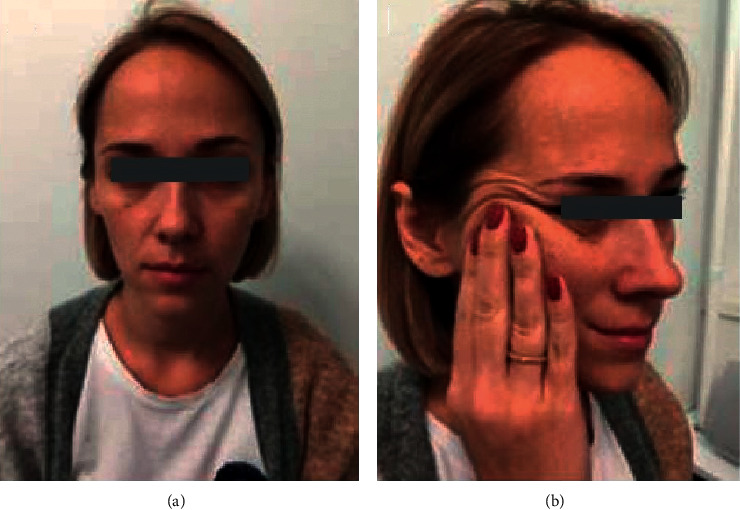
Before correction: facial asymmetry, nasolacrimal, and nasolabial sulci are deeper on the right (a); skin is easy folded (b).

The patient, 36 years old, complained on severe asymmetry of the face, infraorbital, and nasolabial sulci, and thin and easily folding skin (Figure 1).

### 4.2. History of the Disease

Facial changes appeared after 22 years of age. The following CTD criteria were met based on the patient's answers: mitral valve prolapse, varicose veins, moderate myopia, poor posture (scoliotic spinal deformity), joint hypermobility, and pelvic prolapse after childbirth.

### 4.3. Clinical Evidence

Examination: sluggish, thin, easily stretchable (more than 3 cm) skin. Wrinkle scores as assessed by the Lemperle scale: nasolabial folds: 2 points; infraorbital sulci: 2 points.

The severity of facial skin changes was also assessed by the “TPGAZh RMK” system, where “T” parameter defines the tone (turgor + hydration + elasticity) of the skin:

- T(1)—normotonic (turgor preserved)
- T(2)—hypotonic (turgor reduced)
- T(3)—atonic (turgor loss, significant)

“P” parameter indicates the severity of facial cutaneous ptosis:

- P(0)—no ptosis
- (1)—ptosis in the buccal area
- P(2)—ptosis in the submandibular area
- P(3)—ptosis over the entire facial surface, to the neck

“G” parameter assesses skin pigmentation:

- G(0)—no pigmentation
- G(1)—local hypopigmentation
- G(2)—local hyperpigmentation
- G(3)—hyperpigmentation of the entire facial surface, to the neck

“A” parameter characterizes the muscular aponeurotic structure of the face and neck:

- А(0)—no weakness of the muscular aponeurotic structure
- А(1)—local weakness of the muscular aponeurotic structure
- А(2)—weakness of the muscular aponeurotic structure over the facial surface
- А(3)—severe weakness over the entire facial surface to the neck

“Zh” parameter assesses the adipose tissue:

- Zh(0)—poorly developed
- Zh(1)—regular consistency, evenly distributed over the face
- Zh(2)—regular consistency with local deposits
- Zh(3)—loose, with elevated deposits in the buccal and submandibular areas, spread to the neck

“R” parameter assesses the mimics:

- R(0)—no mimic facial expressions
- R(1)—poor mimic facial expressions
- R(2)—many mimic facial expressions
- R(3)—intense mimic facial expressions (tics)

“M” parameter indicates wrinkle severity:

- M(0)—no wrinkles
- M(1)—grade I wrinkles
- M(2)—grade II wrinkles
- M(3)—grade III wrinkles

“K” parameter defines the skin type:

- K(1)—normal skin
- K(2)—dry skin
- K(3)—oily skin [21]

### 4.4. Clinical Conclusion

The baseline condition of the facial superficial soft tissues was assessed using the above system and found corresponding to the II grade age-related changes: T2-3P1G0A2Zh1 R1M2K2.

### 4.5. Aim of Correction

- Improved quality of the facial skin and soft tissues
- Elimination of asymmetry
- Decreased depth of sulci

### 4.6. Method of Correction

Based on the medical history, patient's complaints, and physical examination data, collagen stimulator CaHA-based product diluted 1 : 1 with the normal saline was chosen for skin correction in the middle and lower facial thirds.

### 4.7. Procedure

Application anesthesia with EMLA medicinal product was used on insertion points. Prior to administration, 1.5 mL of the CaHA-based product was combined with 0.25 mL of 2% lidocaine in accordance with the established standards. Then, 1.75 mL of the normal saline NaCl was added to 1.75 mL of the resulting product through the connector and mixed to homogeneity (not less than 10 passes). A 25G × 5 cm cannula was used for injection. The needle insertion points were chosen according to the French consensus scheme [22] at the mental fold and the zygomatic arch border in the area of the midpalatal sulcus. The skin was punctured with a 21G needle to insert the cannula into the subdermal layer.

The prepared product was injected using the radial technique into the upper layers of the dermis and into the subdermal layer. After the procedure, the injected product was distributed manually in the area of correction. Immediately after the procedure, no ecchymoses were noted, and mild posttraumatic edema was observed. An agreement on the participation in this study and consent to the provision of photos before and after the cosmetic procedures with the necessary level of anonymity, which caused partial concealment of the face on the photo, was signed with the patient. At the same time, the anonymity of the patient's identity was respected.

## 5. Results

**Figure 2:**
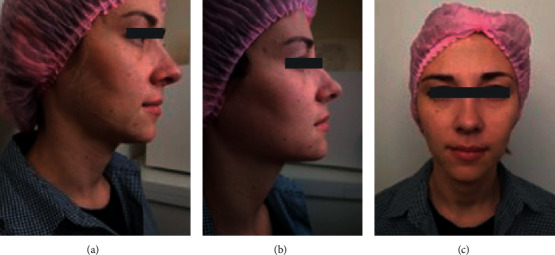
After one procedure (a), after two procedures with calcium hydroxylapatite injection (1.5 mL) (b, c).

Result after the 1st treatment procedure. Visually, the face looked younger, and fast lifting of the facial soft tissues is visible (Figure 2(a)). After 1.5 months, the treatment procedure was repeated. Result after the 2nd treatment procedure. Both the patient and the physician noted tissue tightening, a significant elimination of asymmetry, and smoothing of infraorbital and nasolabial sulci (Figures 2(b) and 2(c)). After two procedures, the condition of the facial superficial soft tissues was described as T1-2P0G0A1Zh1 P1M1K1 corresponding to the grade I age-related changes in the superficial soft tissues. The quality of correction was rated 3 by the physician, and the level of satisfaction was rated 3 by the patient (ratings on the GAIS, Table 2).

The obtained results demonstrated the efficiency of the applied complex of procedures. At the same time, more tests on more patients are required for wider application.

## 6. Discussion

It has been shown that, in conditions of cosmetology clinic, the correction of facial soft tissues in patients with the diagnosis of early connective tissue dysplasia is possible. Thus, provided a properly chosen therapy strategy and cosmetic procedures, a visit to a cosmetologist is among the mandatory actions in the early stages of connective tissue dysplasia. At the same time, for patients with dysplasia of later stages, medical treatment is mandatory since cosmetic procedures can only give a local or masking effect but do not fundamentally change the metabolic processes occurring in the body, as is possible in the conditions of clinics [23].

After treatment, any complaints from the patient were registered, which also indicates the success of the cosmetic procedures. Performed studies demonstrate the effectiveness of calcium hydroxyapatite application in dysplasia [24]. However, it is necessary to create a database indicating the number of cases of successful or unsuccessful therapy to make conclusions about the effectiveness of the chosen strategy. Statistical analysis of such data would show the probability of a successful outcome in cosmetic procedures depending on the age or gender group of the patient.

The success of modern cosmetology has determined the increasing demands of CTD patients to their appearance. The appearance of patients with dysplasia is known to cause certain inconveniences, depending on the severity of the disease [25]. This leads to the deterioration of interpersonal relations, as well as a decrease in the social adaptation of patients. It should also be noted that, as a rule, patients with dysplasia and different forms of tissue deformation are registered with specialists of different profiles, both doctors and beauticians. All these specialists do not always agree on the selected therapy, which may lead to negative results and worsen the patient's condition.

Women suffer most from the defects caused by dysplasia, as they monitor their appearance, and its negative changes can cause depression and anxiety disorder or a feeling of constant stress. In this condition, women usually go to beauty salons to hide the defects of their appearance [26]. However, the approach aimed at hiding the defects is wrong because it does not cause a therapeutic effect [27]. It has been demonstrated that at a mild CTD, a positive effect is possible when applying calcium hydroxyapatite treatment in the cosmetology salon.

The negative effect is primarily manifested in premature aging. According to some data, the age of premature aging can reach up to nine years when comparing patients from the control group without dysplasia and patients from the group diagnosed with dysplasia [28]. Most dysplasia occurs in adolescents. At this age, dysplasia-induced abnormalities are manifested to the maximum extent. From the age of 35, the risk of dysplasia development is sharply reduced. As a rule, disease manifestations at this age are complications caused by dysplasia already existing since adolescence. Complications in adulthood can lead to disability [20]. Thus, monitoring the prevalence of different forms of dysplasia is desirable, including among visitors to beauty salons.

## 7. Conclusions

Connective tissue dysplasia is often associated with pathological changes in the facial soft tissues and early appearance of skin aging signs. It is noteworthy that a CaHA-based product is effective, and the aesthetic effect is durable in patients with CTD. While collecting medical history, it is recommended to take due account of the diagnostic criteria for the connective tissue dysplasia, and, once they are met, to perform the cosmetic correction using collagen stimulators as soon as possible. The performed therapy with the calcium hydroxyapatite injections showed high efficiency on the example of the above medical case. In the future, more patients are planned to be examined in order to recommend the chosen therapy strategy for connective tissue dysplasia. In case when treatment is neglected, significant changes in the mental and physical condition of patients are possible. Therefore, cosmetic procedures aimed not at hiding, but treatment and correction of dysplasia seem to be relevant today.

## Figures and Tables

**Table 1:** Developmental abnormalities corresponding to CTD-related cosmetic syndrome [9].

Signs	Frequency (%)
Morton's toe (the 2nd toe is longer than the 1st)	52.0
Sandal cleft in foot (distance between the 1st and the 2nd toes is ≥ the width of the 2nd toe)	52.0
Compromised dentition	41.0
Clinodactyly (curvature of a digit)	26.0
Third type of earlobe (adherent earlobe)	22.0
Malocclusion	22.0
Low hair growth on the forehead and neck	19.0
Extra teeth	14.5
Heterochromia iridis	11.5
Tongue-tie	10.5
Low-set ears	8.0
Diastema (wide gap between the central incisors)	8.0
Upper lip frenulum	8.0
Deformed ears	7.5
Hypotelorism (close-set eyes)	7.0
Big sticking-out ears	7.0
Epicanthic fold (skinfold in the medial canthus)	6.5
Exophthalmos	6.5
Enophthalmos	6.0
Ocular hypertelorism (increased distance between the inner eye corners)	5.0
Crumpled-ear deformity	5.0
Nipple hypertelorism	4.5
Camptodactyly (contracture of the proximal interphalangeal joint)	2.0
Brachydactyly	1.5
Polythelia (more than 2 nipples)	1.0

**Table 2:** Ratings on the global aesthetic improvement scale (GAIS).

Score	Physician rating	Patient rating
3	Optimal cosmetic result for this patient	Fully satisfied with the treatment result
2	Marked improvement, but not completely optimal	The patient is satisfied with the treatment result, but still wants to improve it
1	Improvement of the appearance, better compared with the initial condition, but a touch-up is advised	Insignificant improvement, a touch-up is advised
0	No changes	No changes
−1	The appearance has worsened compared with the original condition	The appearance has worsened compared with the condition before treatment

## Data Availability

The data used to support the findings of this study are available from the corresponding author upon request.
